# Phase I studies of vorinostat with ixazomib or pazopanib imply a role of antiangiogenesis-based therapy for *TP53* mutant malignancies

**DOI:** 10.1038/s41598-020-58366-z

**Published:** 2020-02-20

**Authors:** Yudong Wang, Filip Janku, Sarina Piha-Paul, Kenneth Hess, Russell Broaddus, Lidong Liu, Naiyi Shi, Michael Overman, Scott Kopetz, Vivek Subbiah, Aung Naing, David Hong, Apostolia M. Tsimberidou, Daniel Karp, James Yao, Siqing Fu

**Affiliations:** 1 Departments of Investigational Cancer Therapeutics, The University of Texas MD Anderson Cancer Center, Houston, TX USA; 2 Departments of Biostatistics, The University of Texas MD Anderson Cancer Center, Houston, TX USA; 3 Departments of Pathology, The University of Texas MD Anderson Cancer Center, Houston, TX USA; 4 Departments of GI Medical Oncology, The University of Texas MD Anderson Cancer Center, Houston, TX USA; 5 Department of Medical Oncology, The Fourth Hospital of Hebei Medical University, Hebei, People’s Republic of China

**Keywords:** Predictive markers, Phase I trials, Targeted therapies

## Abstract

We performed two phase I trials of the histone deacetylase inhibitor vorinostat combined with either the vascular endothelial growth factor inhibitor pazopanib (NCT01339871) or the proteasome inhibitor ixazomib (NCT02042989) in patients with metastatic *TP53* mutant solid tumors. Both trials followed a 3 + 3 dose-escalation design allowing for a dose expansion cohort of up to 14 additional patients with a specific tumor type. Patients had to have a confirmed *TP53* mutation to be enrolled in NCT02042989. Among patients enrolled in NCT01339871, TP53 mutation status was determined for those for whom tumor specimens were available. The results of NCT01339871 were reported previously. Common treatment-related adverse events in NCT02042989 included anemia, thrombocytopenia, fatigue, nausea, vomiting, and diarrhea. Compared with patients with metastatic *TP53* hotspot mutant solid tumors who were treated with ixazomib and vorinostat (n = 59), those who were treated with pazopanib and vorinostat (n = 11) had a significantly higher rate of clinical benefit, defined as stable disease lasting ≥6 months or an objective response (3.4% vs. 45%; *p* < 0.001), a significantly longer median progression-free survival duration (1.7 months [95% confidence interval (CI), 1.1–2.3] vs. 3.5 months [95% CI, 1.7–5.2]; *p* = 0.002), and a longer median overall survival duration (7.3 months [95% CI, 4.8–9.8] vs. 12.7 months [95% CI, 7.1–18.3]; *p* = 0.24). Our two phase I trials provide preliminary evidence supporting the use of antiangiogenisis-based therapy in patients with metastatic *TP53* mutant solid tumors, especially in those with metastatic sarcoma or metastatic colorectal cancer.

## Introduction

In advanced cancers and those refractory to treatment, many factors may play a contributing role. Tumor cells that survive antiangiogenic therapy and metastasize frequently do so in hypoxic microenvironments^1–3^. Tumor hypoxia upregulates histone deacetylase (HDAC) activity^4^, which modulates the overexpression of hypoxia-inducible factor 1α (HIF-1α)^5^. This hypoxia-mediated increase in HIF-1α promotes tumor progression through the HIF-1α−dependent activation of multiple genes, whose expression enables cancer cells to survive, metastasize, and acquire resistance to antiangiogenic therapy^6,7^. HDAC5 is a critical player in the p53 acetylation network^8,9^, and HDAC6 and HDAC8 interact with heat shock protein 90 to facilitate mutant p53 degradation^10–12^. HDAC inhibition with vorinostat preferentially kills *TP53* mutant cancer cells in cell cultures and xenograft models^10,11^.

The enhanced vascular endothelial growth factor (VEGF) pathway plays an important role in the survival and proliferation of cancer cells with *TP53* mutations^13,14^ and thus represents a potential therapeutic target in *TP53* mutant cancers. In cancer cells, *TP53* mutations are associated with elevated HIF-1α levels, which augment the HIF-1α−dependent transcriptional activation of the *VEGF* gene in response to tumor hypoxia^15^, and mediate resistance to cancer therapy^16^. In addition, we found that among cancer patients receiving VEGF inhibition−based therapies, the progression-free survival (PFS) durations of patients with mutated *TP53* were significantly longer than those of patients with wild-type *TP53*^16–19^.

The ability of wild-type p53 protein to induce apoptosis and suppress angiogenesis is of significant scientific merit and urgent clinical interest to develop novel cancer therapeutics. One promising strategy to explore HDAC inhibitor-mediated down-regulation of HIFs for targeting *TP53* mutant tumor resistance to antiangiogenic therapy is supported by both preclinical and retrospective clinical findings^20–27^. To date, the U.S. Food and Drug Administration has approved pazopanib for the treatment of renal cell carcinoma and soft tissue sarcoma; vorinostat for the treatment of primary cutaneous T-cell lymphoma; and ixazomib for the treatment of multiple myeloma. We therefore conducted two phase I trials: one of the HDAC inhibitor vorinostat plus the VEGF inhibitor pazopanib in patients with advanced malignancies (NCT01339871) and another of vorinostat plus the proteasome inhibitor ixazomib in patients with metastatic *TP53* mutant solid tumors (NCT02042989).

## Results

### Patient characteristics

The characteristics of the 78 patients enrolled in the phase I trial of pazopanib and vorinostat were reported previously^28^. The characteristics of the 59 patients enrolled in the phase I trial of ixazomib and vorinostat are given in Table 1. The phase I trial of ixazomib and vorinostat followed a 3 + 3 dose-escalation design. Patients were enrolled at 4 dose levels. One treatment cycle was 28 days. Oral ixazomib, escalating from 3 to 4 mg, was administered on days 1, 8, and 15, and oral vorinostat, escalating from 100 mg twice daily to 100 mg three times daily, was given on days 1–21. The patients enrolled in the ixazomib and vorinostat trial, whose median age was 59 years (range, 24−76 years), were heavily pretreated; they received a median of 5 systemic therapeutic regimens previously, and 58% had experienced disease progression on VEGF inhibition−based therapy.

**Table 1:** Characteristics of patients with confirmed *TP53* mutations.

Characteristic	Clinical Trial	
	Ixazomib + Vorinostat (n = 59)	Pazopanib + Vorinostat (n = 11)
Median age (range), years	59 (24–76)	70 (46–78)
Gender		
Male	24 (41)	5 (45)
Female	35 (59)	6 (55)
Race		
White	46 (78)	9 (82)
Hispanic	5 (8)	0
African American	4 (7)	0
Asian	4 (7)	2 (2)
ECOG performance status score		
0	11 (19)	0
1	44 (74)	11 (100)
2	4 (7)	0
Disease type		
Colorectal cancer	20 (34)	3 (27)
Ovarian cancer	14 (23)	3 (27)
Breast cancer	4 (7)	1 (9)
Sarcoma	4 (7)	2 (18)
Head and neck cancer	4 (7)	1 (9)
Others*	13 (22)	1 (9)
Prior chemotherapy		
Median no. of regimens (range)	5 (0–9)	4 (0–10)
VEGF inhibition−based therapy	34 (58)	5 (45)
Prior radiation therapy	33 (56)	3 (27)
Prior surgery	47 (80)	10 (91)
Median no. of metastasis sites (range)	3 (1–5)	3 (2–5)
*TP53* point mutations	50 (85)	9 (82)
*TP53* hotspot mutations^#^	24 (41)	4 (36)
*TP53* non-point mutations	9 (15)	2 (18)

Note: All data are no. of patients (%) unless otherwise noted.

Abbreviations: ECOG, Eastern Cooperative Oncology Group; VEGF, vascular endothelial growth factor.

*Includes duodenal, gastric, and pancreatic cancer (n = 2 each) and esophageal cancer, endometrial cancer, non-small cell lung cancer, renal cancer, urachal adenocarcinoma, melanoma, and Mullerian tumor (n = 1 each).

^#^Mutations at R175, G245, R248, R249, R273, or R282.

### Safety evaluation

In the phase I trial of pazopanib and vorinostat, the recommended phase II dosage was 600 mg pazopanib daily in combination with 100 mg vorinostat three times daily^28^. In the phase I trial of ixazomib and vorinostat, the recommended phase II dosage was 4 mg ixazomib once daily on days 1, 8, and 15 in combination with 100 mg vorinostat three times daily on days 1−21 (dose level 4). The clinically significant grade 2 or higher adverse events experienced by patients treated with ixazomib and vorinostat included anemia, thrombocytopenia, fatigue, anorexia, nausea, vomiting, diarrhea, dehydration, and skin rash (Supplementary Table 1). No treatment-related death or dose-limiting toxicity was observed among these patients. Seven patients, all of whom were enrolled at dose level 4, required dose reductions (18%). The patient withdrawal rates at dose levels 2, 3, and 4 were 13% (1 of 8 patients), 17% (1 of 6 patients), and 31% (12 of 39 patients), respectively.

### Efficacy evaluation

#### Antitumor activity and survival among all patients

**Figure 1:**
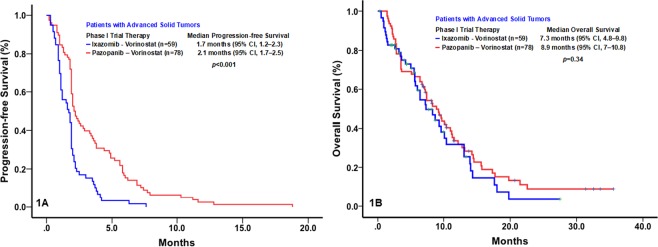
Kaplan-Meier plots of the rates of progression-free survival (PFS; 1A) and overall survival (OS; 1B) of cancer patients treated with pazopanib and vorinostat (n = 78) or ixazomib and vorinostat (n = 59).

The major clinical outcomes of the 59 patients enrolled in the phase I trial of ixazomib and vorinostat are shown in Table 2. No objective responses were observed in these patients. Compared with patients treated with ixazomib and vorinostat, those treated with pazopanib and vorinostat had a significantly higher rate of clinical benefit, defined as stable disease lasting ≥6 months, a partial response, or a complete response (3.4% vs. 19%; *p* = 0.007) and a significantly longer median PFS duration (1.7 months [95% confidence interval (CI), 1.1–2.3] vs. 2.1 months [95% CI, 1.7–2.5]; *p* < 0.001). However, the median OS durations of the patients treated with ixazomib and vorinostat (7.3 months; 95% CI, 4.8–9.8) and those treated with pazopanib and vorinostat (8.9 months; 95% CI, 7.0–10.8) did not differ significantly (*p* = 0.34) (Fig. 1A,B).

**Table 2:** Major Clinical Outcomes in the Phase I Trial of Ixazomib and Vorinostat (n = 59).

*TP53* mutation	Age	Sex	Race	PS	Pathology	Dose Level	Best response	PFS (mo)	OS (mo)
R273C	43	F	A	1	CRC	1	PD	1.0	3.4+
R273H	41	M	W	0	Gastric	1	PD	1.9	14.6
R175H	64	M	W	1	CRC	1	SD	3.7	7.2
Splice site c.376–1 G > A	59	F	W	1	SA	1	PD	1.9	13.3+
G245S	64	M	AA	1	Pancreatic	1	PD	0.6	1.1
R248W	60	F	W	1	Breast	1	PD	2.0	6.2
R196*	76	F	W	1	EOC	2	PD	1.1	6.3
S127F	67	F	W	1	Breast	2	PD	0.9	2.0
V173M	55	F	W	1	Breast	2	PD	1.6	6.5
H179P	58	M	H	0	CRC	2	SD	3.0	15.0
Q104*	52	M	W	1	HNC	2	Withdrawal	0.3	1.3+
G245S	56	F	W	2	Urachal	2	PD	2.1	18.2
R342*	40	M	W	0	CRC	2	PD	1.9	13.7
V10I	31	M	W	1	SA	2	PD	1.2	10.7+
L111P	64	M	W	2	Gastric	3	PD	1.0	9.7
R280T	53	F	H	1	Breast	3	SD	3.6	7.2
R213L	75	M	W	1	CRC	3	PD	2.0	29.2+
A138V	60	M	W	1	CRC	3	PD	1.0	1.7
V272L	42	M	W	1	CRC	3	SD	3.5	14.6
L201*	64	M	W	1	CRC	3	Withdrawal	2.3	19.9
P278A	63	F	W	1	EOC	4	Withdrawal	1.2	4.7
G245S	38	M	AA	1	CRC	4	PD	0.9	2.1
C135R	70	F	W	1	Endometrial	4	PD	1.0	14.1
S215N	57	M	W	1	HNC	4	PD	1.8	8.4
S241F	51	M	AA	1	NSCLC	4	PD	2.2	5.2
R282W	60	M	W	1	CRC	4	Withdrawal	1.6	3.1
R248W	60	F	W	1	CRC	4	PD	1.9	9.1
R280G	70	F	W	2	EOC	4	SD	7.6	10.2
R175H	52	F	W	0	CRC	4	Withdrawal	1.1	7.4
R248Q	35	M	W	1	Melanoma	4	PD	0.7	4.4
R213*	48	F	W	1	CRC	4	PD	1.9	10.8
C176S, R248Q, R273H	71	M	W	0	SA	4	SD	3.9	18.3
G244S	66	F	H	1	CRC	4	PD	1.5	10.4
R273C	60	M	W	1	CRC	4	PD	1.1	2.5
E336Q/ Y234H	52	M	A	1	CRC	4	Withdrawal	0.9	5.8
C229fs*10	64	F	H	1	EOC	4	SD	6.3	8.6
E180fs*67	58	F	W	1	EOC	4	PD	2.1	5.1
R248W	33	F	W	1	CRC	4	Withdrawal	0.6	7.0
Y205H	76	M	W	1	CRC	4	PD	0.5	2.4
Q16*/ R248Q	63	F	W	1	HNC	4	PD	0.3	1.1
R282W	58	F	W	1	Mullerian	4	SD	4.1	11.3
Y220C	73	F	AA	2	EOC	4	Withdrawal	0.9	2.8+
R175H	50	F	H	1	EOC	4	PD	1.9	4.9+
G245R/ K164E	50	F	W	0	EOC	4	SD	3.8	10.8+
R282W	24	F	W	1	EOC	4	Withdrawal	0.7	2.2
H179Y	67	F	W	0	EOC	4	PD	1.8	8.9+
R306*	42	M	W	1	Renal	4	PD	2.2	7.9+
C275F	64	F	A	1	CRC	4	Withdrawal	0.5	6.3+
R273C	68	F	A	0	Pancreatic	4	PD	1.8	2.5+
R175H	56	F	W	1	Cecum	4	Withdrawal	0.9	3.7
R110L	60	F	W	0	EOC	4	PD	1.9	6.6+
L111P	73	F	W	1	EOC	4	Withdrawal	1.7	6.3+
H179R/R273C	59	F	W	1	EOC	4	Withdrawal	1.2	2.8+
R282W	46	F	W	1	CRC	4	SD	4.2	7.2+
R273H	58	M	W	1	Cecum	4	Lost to follow up	1.1	2.8
R273H	57	F	W	0	HNC	4	Withdrawal	0.3	5.7+
Y234N/R158G	61	M	W	0	Esophageal	4	PD	1.9	4.6+
V173L	57	F	W	1	SA	4	PD	2.5	3.3+
P278_G279del	66	F	W	1	EOC	4	PD	1.1	3.5+

Abbreviations: PS, ECOG performance status; PFS, progression-free survival; OS, overall survival; mo, month; M, male; F, female; W, white; A, Asian; H, Hispanic; AA, African-American; PD, progression disease; SD, stable disease; + , censored; CRC, colorectal cancer; EOC, epithelial ovarian cancer; SA, sarcoma; HNC, head & neck cancer; NSCLC, non-small cell lung cancer.

#### Antitumor activity and survival among patients with tp53 hotspot mutations

**Figure 2:**
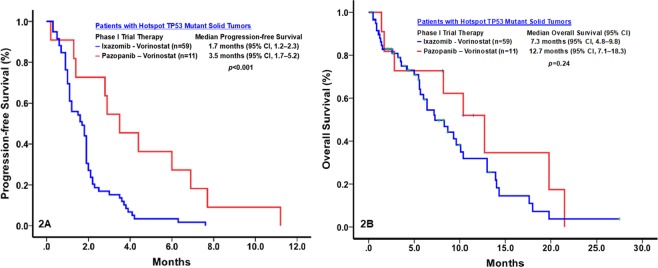
Kaplan-Meier plots of the rates of progression-free survival (PFS; 2A) and overall survival (OS; 2B) of patients with metastatic *TP53* mutant solid tumors treated with pazopanib and vorinostat (n = 11) or ixazomib and vorinostat (n = 59).

*TP53* hotspot mutations were confirmed in all 59 patients enrolled in the phase I trial of ixazomib and vorinostat and in 11 of the 78 patients enrolled in the phase I trial of pazopanib and vorinostat. Compared with patients treated with ixazomib and vorinostat, those treated with pazopanib and vorinostat had a significantly higher rate of clinical benefit (3.4% vs. 45%; *p* < 0.001) and a significantly longer median PFS duration (1.7 months [95% CI, 1.1–2.3] vs. 3.5 months [95% CI, 1.7–5.2]; *p* = 0.002). The median OS duration of the patients treated with pazopanib and vorinostat (12.7 months; 95% CI, 7.1–18.3) was longer than that of those treated with ixazomib and vorinostat (7.3 months; 95% CI, 4.8–9.8), but this difference was not significant (*p* = 0.24) (Fig. 2A,B).

#### Antitumor activity and survival among patients with metastatic sarcoma or colorectal carcinoma with TP53 hotspot mutations

**Figure 3:**
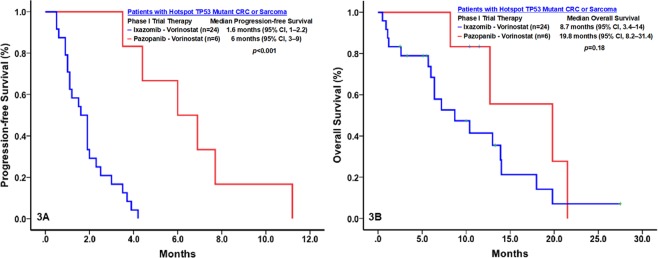
Kaplan-Meier plots of the rates of progression-free survival (PFS; A) and overall survival (OS; B) of patients with metastatic *TP53* mutant sarcoma or colorectal cancer (CRC) treated with pazopanib and vorinostat (n = 6) or ixazomib and vorinostat (n = 24).

Twenty-four patients enrolled in the phase I trial of ixazomib and vorinostat had colorectal carcinoma (n = 20) or sarcoma (n = 4) with *TP53* hotspot mutations, and 6 patients enrolled in the phase I trial of pazopanib and vorinostat had colorectal carcinoma (n = 3) or sarcoma (n = 3) with *TP53* hotspot mutations. Compared with patients treated with ixazomib and vorinostat, those treated with pazopanib and vorinostat had a significantly higher rate of clinical benefit (0% vs. 83%; *p* < 0.001) and a significantly longer median PFS duration (1.6 months [95% CI, 1–2.2] vs. 6.0 months [95% CI, 3.0–9.0]; *p* < 0.001). The median OS duration of the patients treated with pazopanib and vorinostat (19.8 months, 95% CI, 8.2–31.4) was longer than that of those treated with ixazomib and vorinostat (8.7 months, 95% CI, 3.4–14), but this difference was not significant (*p* = 0.18) (Fig. 3A,B).

## Discussion

Therapeutic targeting of *TP53* mutations is a rapidly developing field, and various approaches have undergone clinical evaluation^29,30^. We have designed and conducted two subsequent phase I clinical trials (NCT01339871, a phase I study of pazopanib and vorinostat in patients with advanced malignancies; and NCT02042989, a phase I study of ixazomib and vorinostat in patients with advanced *TP53* mutant malignancies) in order to target patients with metastatic *TP53* mutant solid tumors. Our two phase I clinical trials provide preliminary prospective evidence supporting the use of antiangiogenesis-based therapy in patients with *TP53* mutations.

Our phase I trial of pazopanib and vorinostat revealed that the combination therapy was more effective in cancer patients with *TP53* hotspot mutations than in patients without TP53 hotspot mutations^28^. Many cancers have *TP53* mutations^31^, many of which produce mutant p53 gain-of-function proteins, leading to tumorigenesis, tumor development, and metastasis^32,33^. Because they promote tumor growth through VEGF overexpression and increased neovascularization^34^ and regulate cell cycle arrest, DNA damage repair, cellular senescence, apoptosis, metabolism, stem cell maintenance, tumor invasion, metastasis, and communication with the tumor microenvironment^35–37^, mutant p53 proteins are potential therapeutic targets^16,38^. To be effective against *TP53* mutant malignancies, a therapy must target many biological pathways simultaneously. The results of our phase I trial of the HDAC inhibitor vorinostat plus the VEGF inhibitor pazopanib support the use of this combination in patients with *TP53* mutant malignancies^28^. These agents likely have antitumor activity through their synergistic antiangiogenic effects, facilitation of mutant p53 degradation^10–12^, and downregulation of VEGF inhibition−mediated HIF-1α overexpression^5,15,27^.

Preclinical studies have shown that proteasome inhibition induces p53-dependent and -independent apoptosis and that HDAC inhibition mediates preferential cytotoxicity towards p53 mutant cells. In addition, combined proteasome and HDAC inhibition has synergistic antitumor effects by modulating epigenetic gene expression^39,40^, posttranslational modifications^41^, and protein degradation in the proteasome and aggresome pathways^42,43^, thereby increasing cellular stress and apoptosis^44^. On the basis of these findings, we conducted a phase I trial of ixazomib and vorinostat in 59 patients with metastatic *TP53* mutant solid tumors, expecting to find that the combination had efficacy against these tumors. However, the combination did not elicit an objective response in any of these patients and was associated with poor PFS and OS. These findings led us to initiate the present study to investigate the role of these therapeutic regimens in patients with *TP53* mutant malignancies by comparing their antitumor activity and associated survival outcomes between the two phase I trials. Among patients with metastatic solid tumors with *TP53* hotspot mutations – particularly patients who had colorectal carcinoma or sarcoma – those treated with pazopanib and vorinostat had a significantly longer median PFS duration and a longer OS duration than did those treated with ixazomib and vorinostat. These findings suggest that vorinostat should be paired with pazopanib, rather than ixazomib, for the treatment of cancers with *TP53* mutations.

The present study yielded several additional insights into the use of vorinostat with pazopanib or ixazomib against metastatic solid tumors with *TP53* mutations. First, although preclinical evidence suggests that HDAC inhibition induces preferential cytotoxicity towards p53 mutant cells, our findings suggest that the use of vorinostat most likely contributed to the high frequencies of dose reduction and patient withdrawal owing to drug toxicity in the two trials. Thus, additional studies to determine whether lower doses of HDAC inhibitors can have meaningful therapeutic effect against p53 mutant cancer cells may be warranted. Second, many patients can be maintained at lower dose levels. There is no statistical relationship between dose level and major clinical outcomes including antitumor responses and survivals. Third, because p53 is at the hub of numerous signaling pathways triggered by a range of cellular stresses, effective therapeutic strategies against malignancies driven by p53 mutations may require several agents simultaneously targeting multiple p53-regulated downstream pathways.

The present study had several potential limitations that could limit the clinical relevance of its findings. First, as with many early clinical trials, the phase I trials we conducted—despite employing eligibility criteria similar to those used in other phase I trials enrolling patients with advanced solid tumors lacking effective standard therapy—were subject to patient selection bias, which may limit the generalizability of our findings to patients with *TP53* mutant malignancies. Second, owing to their small sample sizes, the subgroup analyses could not reliably detect significant differences between groups. Third, because they did not include correlative pharmacokinetics and pharmacodynamics assessments, the phase I trials may not have identified the optimal recommended phase II doses of the drug combinations they tested.

## Patients and Methods

### Trial design and patient enrollment

The trial of pazopanib and vorinostat enrolled patients with metastatic solid tumors from April 2011 to December 2013, whereas the trial of ixazomib and vorinostat enrolled patients with metastatic solid tumors carrying *TP53* hotspot mutations, defined as positive cytoplasmic staining by immunohistochemistry and/or next gene mutation sequencing from July 2014 to February 2017. For both trials, patients were age ≥18 years, with a histologically confirmed advanced malignancy and without a standard therapy that improved survival ≥3 months. All eligible patients also had measurable or evaluable disease that had progressed prior to enrollment and an Eastern Cooperative Oncology Group performance status score of ≥2^45^. Additional eligibility criteria included adequate marrow function (absolute neutrophil count ≥1,000/μl and platelet count ≥75,000/μl), a calculated creatinine clearance rate of ≥30 ml/min, a total bilirubin level ≤1.5 × the upper limit of the normal (ULN), and alanine aminotransferase and aspartate aminotransferase levels ≤3 × the ULN. Patients were excluded if they had clinically significant cardiovascular disease; had active uncontrolled central nervous system involvement; had active serious infection requiring systemic antibiotics; had known gastrointestinal disease or other condition that could interfere with swallowing or oral absorption; were pregnant or lactating; had not recovered from previous cancer therapeutics; or were unwilling or unable to give written informed consent. Both trials were conducted at MD Anderson and were approved by MD Anderson’s Institutional Review Board. Informed consent was obtained from all study participants, and all methods were performed in accordance within the relevant guidelines and regulations.

### Evaluation of toxicity and efficacy

Patients who had received at least one dose of any of the study agents were considered evaluable for drug safety. Toxicity severity was graded according to the Common Terminology Criteria for Adverse Events, version 4.0 (http://ctep.cancer.gov/protocolDevelopment/electronic_applications/ctc.htm#ctc_40). Dose-limiting toxicity was defined as treatment-related grade 4 hematologic toxicity lasting >1 week; grade 4 nausea or vomiting lasting >3 days despite appropriate medical intervention; grade 4 fatigue or hypertension; or grade 3 or higher non-hematologic toxicity occurring within the initial 28-day treatment cycle. The maximum tolerated dose was defined as the dose level below that at which >33% of patients experienced dose-limiting toxicity.

Patients who received at least one dose of any of the study agents were considered evaluable for drug efficacy. Patients receiving therapy underwent radiographic imaging studies every 8 weeks. We used Response Evaluation Criteria in Solid Tumors 1.1 to assess tumor responses^46^.

### Molecular assays for *TP53* mutations

Archival formalin-fixed, paraffin-embedded tissue blocks, core biopsy specimens, or surgical resection specimens were used for *TP53* mutation assessment. *TP53* mutation assessment was performed in a Clinical Laboratory Improvement Amendment−certified molecular diagnostic laboratory at MD Anderson Cancer Center (for hotspots 2 [1–20], 4 [68–113], 5 [126–138], 5 [149–187], 6 [187–223], 7 [225–258], 8 [263–307], and 10 [332–367]), as well as at Foundation Medicine (for the entire coding sequence), as described previously^47–49^.

### Statistical considerations

Both phase I trials followed a modified zone-based 3 + 3 dose-escalation design^50^. An additional cohort of up to 3 patients was allowed per dose level as needed for safety assessment. If clinical benefit was observed in patients with a specific type of cancer, a dose expansion cohort of up to 14 patients was permitted at the highest dose level considered to be safe at the time of patient entry, as described previously^51^. Continuous data were summarized using medians, ranges, and 95% CIs. Categorical data were summarized using frequencies and percentages. Differences in categorical variables were assessed using the Fisher exact test. PFS was defined as the time from the date of initial trial therapy (cycle 1 day 1) to the date of death or tumor progression. OS was defined as the time from the date of initial trial therapy to the date of death or last radiographic assessment. Patients who had no evidence of disease progression or were alive at the end of the study period were censored at the date of last radiographic assessment. The Kaplan-Meier method was used to estimate PFS and OS, and log-rank tests were used to compare PFS and OS distributions between the treatment groups. Differences between the treatment groups were assessed using two-sided t-tests; *p* values < 0.05 were considered significant. Statistical analyses were performed using SPSS Statistics, version 24 (IBM, Armonk, NY).

## Supplementary information

Supplementary information

## References

[1] Paez-Ribes M (2009). Antiangiogenic therapy elicits malignant progression of tumors to increased local invasion and distant metastasis. Cancer Cell.

[2] Ebos JM (2009). Accelerated metastasis after short-term treatment with a potent inhibitor of tumor angiogenesis. Cancer Cell.

[3] Simonsen TG, Gaustad JV, Rofstad EK (2010). Development of hypoxia in a preclinical model of tumor micrometastases. Int. J. Radiat. Oncol. Biol. Phys..

[4] Fath DM (2006). Histone deacetylase inhibitors repress the transactivation potential of hypoxia-inducible factors independently of direct acetylation of HIF-alpha. J. Biol. Chem..

[5] Kim SH (2007). Regulation of the HIF-1alpha stability by histone deacetylases. Oncol. Rep..

[6] Keith B, Simon MC (2007). Hypoxia-inducible factors, stem cells, and cancer. Cell.

[7] Loges S, Mazzone M, Hohensinner P, Carmeliet P (2009). Silencing or fueling metastasis with VEGF inhibitors: antiangiogenesis revisited. Cancer Cell.

[8] Sen N, Kumari R, Singh MI, Das S (2013). HDAC5, a key component in temporal regulation of p53-mediated transactivation in response to genotoxic stress. Mol. Cell.

[9] Marks PA, Richon VM, Miller T, Kelly WK (2004). Histone deacetylase inhibitors. Adv. Cancer Res..

[10] Li D, Marchenko ND, Moll UM (2011). SAHA shows preferential cytotoxicity in mutant p53 cancer cells by destabilizing mutant p53 through inhibition of the HDAC6-Hsp90 chaperone axis. Cell Death Differ..

[11] Yan W, Liu S, Xu E, Zhang J, Zhang Y, Chen X, Chen X (2012). Histone deacetylase inhibitors suppress mutant p53 transcription via histone deacetylase 8. Oncogene.

[12] Blagosklonny MV (2005). Depletion of mutant p53 and cytotoxicity of histone deacetylase inhibitors. Cancer Res..

[13] Joshi H, Bhanot G, Borresen-Dale AL, Kristensen V (2012). Potential tumorigenic programs associated with TP53 mutation status reveal role of VEGF pathway. Br. J. Cancer.

[14] Montero E, Abreu C, Tonino P (2008). Relationship between VEGF and p53 expression and tumor cell proliferation in human gastrointestinal carcinomas. J. cancer Res. Clin. Oncol..

[15] Ravi R (2000). Regulation of tumor angiogenesis by p53-induced degradation of hypoxia-inducible factor 1alpha. Genes. Dev..

[16] Said R (2014). Characteristics and survival of patients with advanced cancer and p53 mutations. Oncotarget.

[17] Wang Z (2016). Survival of patients with metastatic leiomyosarcoma: the MD Anderson Clinical Center for targeted therapy experience. Cancer Med..

[18] Wang Z (2017). Antiangiogenesis and gene aberration-related therapy may improve overall survival in patients with concurrent KRAS and TP53 hotspot mutant cancer. Oncotarget.

[19] Wang Y (2018). Outcome analysis of Phase I trial patients with metastatic KRAS and/or TP53 mutant non-small cell lung cancer. Oncotarget.

[20] Fraisl P, Mazzone M, Schmidt T, Carmeliet P (2009). Regulation of angiogenesis by oxygen and metabolism. Dev. Cell.

[21] Mazumdar J, Dondeti V, Simon MC (2009). Hypoxia-inducible factors in stem cells and cancer. J. Cell Mol. Med..

[22] Dokmanovic M (2007). Histone deacetylase inhibitors selectively suppress expression of HDAC7. Mol. cancer therapeutics.

[23] Semenza GL (2010). HIF-1: upstream and downstream of cancer metabolism. Curr. Opin. Genet. Dev..

[24] Richardson PG, Anderson KC (2003). Bortezomib: a novel therapy approved for multiple myeloma. Clin. Adv. Hematol. Oncol..

[25] Pili, R. *et al*. Combination of the histone deacetylase inhibitor vorinostat with bevacizumab in pretreated patients with renal cell carcinoma: Safety, efficacy, and pharmacodynamic results. *Genitourinary Cancers Symposium*, Abstract 350 (2010).

[26] Dasari, A. *et al*. A phase I safety and tolerability study of vorinostat (V) in combination with sorafenib (S) in patients with advanced solid tumors, with exploration of two tumor-type specific expanded cohorts at the recommended phase II dose (renal and non-small cell lung carcinoma). *Journal of Clinical Oncology*, 28:15s, Suppl; abstract 2562 (2010).

[27] Ellis L, Hammers H, Pili R (2009). Targeting tumor angiogenesis with histone deacetylase inhibitors. Cancer Lett..

[28] Fu S (2015). Phase I study of pazopanib and vorinostat: a therapeutic approach for inhibiting mutant p53-mediated angiogenesis and facilitating mutant p53 degradation. Ann. Oncol..

[29] Shen L (2012). The fundamental role of the p53 pathway in tumor metabolism and its implication in tumor therapy. Clin. Cancer Res..

[30] Vousden KH, Ryan KM (2009). p53 and metabolism. Nat. Rev. Cancer.

[31] Green DR, Kroemer G (2009). Cytoplasmic functions of the tumour suppressor p53. Nat..

[32] Muller PA, Vousden KH (2014). Mutant p53 in cancer: new functions and therapeutic opportunities. Cancer Cell.

[33] Leroy B (2013). The TP53 website: an integrative resource centre for the TP53 mutation database and TP53 mutant analysis. Nucleic acids Res..

[34] Ambs S (1998). p53 and vascular endothelial growth factor regulate tumor growth of NOS2-expressing human carcinoma cells. Nat. Med..

[35] Yamakuchi M (2010). P53-induced microRNA-107 inhibits HIF-1 and tumor angiogenesis. Proc. Natl Acad. Sci. USA.

[36] Vogelstein B, Kinzler KW (2004). Cancer genes and the pathways they control. Nat. Med..

[37] Bieging KT, Mello SS, Attardi LD (2014). Unravelling mechanisms of p53-mediated tumour suppression. Nat. Rev. Cancer.

[38] Said R (2013). P53 mutations in advanced cancers: clinical characteristics, outcomes, and correlation between progression-free survival and bevacizumab-containing therapy. Oncotarget.

[39] Mitsiades CS (2004). Transcriptional signature of histone deacetylase inhibition in multiple myeloma: biological and clinical implications. Proc. Natl Acad. Sci. USA.

[40] Mitsiades N (2003). Molecular sequelae of histone deacetylase inhibition in human malignant B cells. Blood.

[41] Mitsiades N (2002). Molecular sequelae of proteasome inhibition in human multiple myeloma cells. Proc. Natl Acad. Sci. USA.

[42] Hideshima T, Richardson PG, Anderson KC (2011). Mechanism of action of proteasome inhibitors and deacetylase inhibitors and the biological basis of synergy in multiple myeloma. Mol. Cancer Ther..

[43] Catley L (2006). Aggresome induction by proteasome inhibitor bortezomib and alpha-tubulin hyperacetylation by tubulin deacetylase (TDAC) inhibitor LBH589 are synergistic in myeloma cells. Blood.

[44] Pei XY, Dai Y, Grant S (2004). Synergistic induction of oxidative injury and apoptosis in human multiple myeloma cells by the proteasome inhibitor bortezomib and histone deacetylase inhibitors. Clin. cancer research: an. Off. J. Am. Assoc. Cancer Res..

[45] Oken MM (1982). Toxicity and response criteria of the Eastern Cooperative Oncology Group. Am. J. Clin. Oncol..

[46] Eisenhauer EA (2009). New response evaluation criteria in solid tumours: revised RECIST guideline (version 1.1). Eur. J. Cancer.

[47] Singh RR (2013). Clinical validation of a next-generation sequencing screen for mutational hotspots in 46 cancer-related genes. J. Mol. diagnostics: JMD.

[48] Frampton GM (2013). Development and validation of a clinical cancer genomic profiling test based on massively parallel DNA sequencing. Nat. Biotechnol..

[49] MacConaill LE (2013). Existing and emerging technologies for tumor genomic profiling. J. Clin. oncology: Off. J. Am. Soc. Clin. Oncol..

[50] Huang X, Biswas S, Oki Y, Issa JP, Berry DA (2007). A parallel phase I/II clinical trial design for combination therapies. Biometrics.

[51] Moroney J (2012). Phase I study of the antiangiogenic antibody bevacizumab and the mTOR/hypoxia-inducible factor inhibitor temsirolimus combined with liposomal doxorubicin: tolerance and biological activity. Clin. cancer research: an. Off. J. Am. Assoc. Cancer Res..

