# Supplementary material for: Phase I studies of vorinostat with ixazomib or pazopanib imply a role of antiangiogenesis-based therapy for TP53 mutant malignancies
Source: Sci Rep. 2020 Feb 20;10:3080. doi: 10.1038/s41598-020-58366-z (PMC7033174; doi:10.1038/s41598-020-58366-z)
Supplement: Supplementary file 1 — Supplementary information [file 41598_2020_58366_MOESM1_ESM.docx]

**Table 1. Frequency of ≥ grade 2 treatment-emergent toxicity by dose level in the phase I trial of ixazomib and vorinostat (n=59)**

| **Dose Level*** | **Number of Patients** | **Toxicity Grade** | Anemia | Thrombocytopenia | Fatigue | Nausea | Vomiting | Diarrhea | Constipation | Mucositis | Headache | Back pain | Abdominal pain | GI bleeding | Bowel obstruction | Colitis | Pulmonary infection | Urinary infection | Hematuria | Thromboembolic | Confusion | Anxiety | Anorexia | Dehydration | Syncope | Dyspnea | Pneumonitis | Skin rashes | Hypotension | Hypertension | Increased AST/ALT | Increased bilirubin | Increased creatinine | Hyperkalemia | Hypokalemia | Hyponatremia | Hypoalbuminemia | Hypophosphatemia | Hypercalcemia | Hyperglycemia | Hypomagnesemia | **Dose Reduction** | **Patient Withdrawal** | **Dose-Limiting Toxicity** |
| --- | --- | --- | --- | --- | --- | --- | --- | --- | --- | --- | --- | --- | --- | --- | --- | --- | --- | --- | --- | --- | --- | --- | --- | --- | --- | --- | --- | --- | --- | --- | --- | --- | --- | --- | --- | --- | --- | --- | --- | --- | --- | --- | --- | --- |
| **1** | **6** | **2** | 3 |  | 1 |  |  |  |  | 1 |  | 1 | 1 |  |  |  |  | 1 |  |  | 1 | 1 |  | 1 |  |  |  |  | 1 |  |  | 1 | 1 |  |  | 1 |  |  |  |  |  | 0 | 0 | 0 |
|  |  | **3** | 1 | 2 | 2 | 1 | 1 | 1 |  |  | 1 |  | 1 |  |  |  |  |  |  |  |  |  |  | 1 |  |  |  |  | 1 | 1 |  |  |  |  |  | 1 |  |  |  |  | 1 |  |  |  |
|  |  | **4** |  |  |  |  |  |  |  |  |  |  |  |  |  |  |  |  |  |  |  |  |  |  |  |  |  |  |  |  |  |  |  |  | 1 |  |  |  |  |  |  |  |  |  |
| **2** | **8** | **2** | 1 |  | 1 | 1 | 1 | 1 |  | 1 |  |  |  |  |  |  | 1 |  |  |  | 1 |  |  |  | 1 | 1 |  | 2 |  |  |  |  |  |  |  | 1 |  |  | 1 |  |  | 0 | 0 | 1 |
|  |  | **3** |  |  |  |  |  |  |  |  |  |  |  |  |  |  |  |  |  |  |  |  |  | 1 | 1 | 1 |  |  |  |  |  |  |  |  | 1 |  |  | 1 |  |  |  |  |  |  |
|  |  | **4** |  |  |  |  |  |  |  |  |  |  |  |  |  |  |  |  |  |  |  |  |  |  |  |  |  |  |  |  |  |  |  |  |  |  |  |  | 1 |  |  |  |  |  |
| **3** | **6** | **2** |  |  | 1 | 1 | 3 | 1 | 1 | 1 |  |  |  |  |  |  | 1 |  |  |  |  |  |  | 1 |  |  |  | 1 | 1 |  |  |  |  |  |  | 1 |  | 1 |  |  |  | 0 | 0 | 1 |
|  |  | **3** | 1 |  |  |  |  | 1 |  |  |  |  |  |  |  |  |  |  | 1 |  |  |  |  |  |  |  |  |  |  |  |  |  |  |  | 1 |  |  |  |  |  |  |  |  |  |
|  |  | **4** |  |  |  |  |  |  |  |  |  |  |  |  |  |  |  |  |  |  |  |  |  |  |  |  |  |  |  |  |  |  |  |  |  |  |  |  |  |  |  |  |  |  |
| **4** | **39** | **2** | 3 | 7 | 8 | 4 | 3 | 1 | 3 |  |  | 2 | 3 |  |  |  | 1 |  |  |  |  |  | 2 | 1 |  |  |  | 1 |  |  | 2 | 1 | 4 | 1 | 1 | 3 | 3 |  |  | 1 |  | 7 | 0 | 12 |
|  |  | **3** | 6 | 2 | 1 | 3 | 3 | 1 |  |  |  |  |  | 2 | 1 | 1 | 1 |  |  | 2 | 1 |  |  |  |  | 2 | 1 |  |  |  |  | 1 | 1 |  |  |  |  |  |  |  |  |  |  |  |
|  |  | **4** |  | 4 |  |  |  |  |  |  |  |  |  |  |  |  |  |  |  |  |  |  |  |  |  | 2 |  |  |  |  |  |  |  |  |  |  |  |  |  | 1 |  |  |  |  |

*Each treatment cycle was 28 days. Oral ixazomib was given on days 1, 8, and 15, and oral vorinostat was given on days 1-21. Dose level 1 = ixazomib 3 mg / vorinostat 100 mg BID; 2 = ixazomib 3 mg / vorinostat 100 mg TID; 3 = ixazomib 4 mg / vorinostat 100 mg BID; and 4 = ixazomib 4 mg / vorinostat 100 mg TID.
